# Lea’s Series of Medical Epitomes

**Published:** 1903-01

**Authors:** 


LEA'S SERIES OF MEDICAL EPITOMES. Diseases of the Skin A Manual for Students and Practitioners. By ALFRED SCHALEK, M. D., instructor of Dermatology, Genitourinary, and Venereal Diseases, Rush Medical College, Chicago. Series edited by Victor C. Pedersen, A. M., M D., House Surgeon at the New York Hospital, New York. 12mo, pp. 225. Illustrated with 34 engravings. Lea Brothers & Co., Philadelphia and New York. 1902. [Price, $1.00.]

In this little volume Dr. Schalek has tersely and plainly stated the fundamental principles of dermatology as at present understood. The descriptions of the various diseases follow in the usual order and are brief but clear. Numerous questions are propounded which are calculated to impress upon the reader the salient points relating to each particular affection.

How much demand there may be for such a book it is not easy to conjecture, although the epitomés by Stelwagon and Shamberg have gone beyond their editions,—one or two others having disappeared with the first edition. It might be claimed that such a work is timely since medical epitomés are now in order, and dermatology should, of course, find its place among them, but it is not altogether certain that such a claim can readily be substantiated in the face of the present superfluity in medical literature.

A small work like this may, indeed, to a certain degree, aid the medical student in remembering points relating to a particular disease, or help a busy practitioner in diagnosticating affections and selecting a quick treatment for them. Still, the authors hope it will be distinctly understood that epitomés are never to be substituted for larger textbooks,—a result easier to deprecate than to prevent.

The pictures are copied from Hyde's and Jackson's larger works. While most of them were acceptable when they first appeared they have not been improved upon by their transfer to this smaller work. G.W.W.

